# A Case of Delirium Tremens, with Remarks

**Published:** 1839-02-02

**Authors:** George Mendenhall

**Affiliations:** Cleaveland, Ohio


*A Case of Delirium Tremens, with Remarks.* By G. Mendenhall, M.D., of Cleaveland, Ohio.

*Cleaveland*, (O.) Jan. 24, 1839.

To the Editors of the Medical Examiner.

Gentlemen,—The lecture of Dr. B. H. Coates, on Delirium Tremens, delivered at the Pennsylvania Hospital, January 2d, and reported in your Journal of the 12th inst., has brought to my mind a case which I will relate to you, hoping that it may possess some little interest.

O. F., aged 52, of intemperate habits for some years, was attacked, about the first of March, 1837, with delirium tremens. On the 7th of March, I was called to see him, with a physician who had been some days in attendance; and, as far as I could gather from him, the treatment had consisted of gently opening the bowels, putting a blister on his back, and giving about one grain of opium every three or four hours. At this time there was violent delirium, and the other usual symptoms of delirium tremens, with a *full* and *bounding* pulse. I proposed venesection, which was objected to by the attending physician; and in consequence of this, the case was left to my sole management. I immediately abstracted sixteen ounces of blood from the arm, ordered two grains of opium every two hours, revulsions to the feet, and repeated the blisters between the shoulders.

*March 8th.*—Had rested better the night previous, and slept a *little.* Ordered a continuance of the opium, in doses of three grains every three hours.

*March 9th.*—More delirium; no rest the night previous; constantly harrassed with the idea that the house was falling on him, and with difficulty could be retained in bed: sweat profusely. I increased the opium to four grains every three hours, and in addition sixty drops of laudanum every four hours.

*March 10th.*—Delirium increasing, and the pulse rather flagging. Ordered some cathartic medicine, which operated well, and continued the opium and laudanum.

*March 11th.*—No better, and the patient growing weaker. I then commenced with laudanum ʒij. every half hour, and increased it in quantity, closely watching its effects every hour, until I had given him four and a half ounces of good laudanum, in a period of twelve hours. Even this produced no sensible effect, except it might be to make the pulse a little slower, the delirium continuing about the same. What was then to be done? "The patient must sleep or die." I procured some Cogniac brandy, of a good quality, and at once gave him a gill; in one hour I repeated it; this quieted him slightly. I next gave him ten grains of opium, and in about one hour he fell asleep, which lasted about fourteen hours. When he awoke, his delirium had almost disappeared, and the patient soon recovered.

The above case has interested me: 1st. From the fact that I thought the pulse indicated bleeding, and which was now followed by some cessation of the delirium; but whether it was of any permanent benefit or not, I cannot say. The patient did not, however, die, and the symptoms at the same time were improved. 2d. The great insensibility of the system to opium. 3d. From the sensibility being increased by the use of brandy and the prompt and favourable action of opium after its exhibition.

Yours, with respect,

George Mendenhall.

